# Temperature Sensing in Modular Microfluidic Architectures

**DOI:** 10.3390/mi7010011

**Published:** 2016-01-18

**Authors:** Krisna C. Bhargava, Bryant Thompson, Anoop Tembhekar, Noah Malmstadt

**Affiliations:** 1 Mork Family Department of Chemical Engineering and Materials Science, University of Southern California, Los Angeles, CA 90089, USA; kcbhar@gmail.com; 2 Department of Biomedical Engineering, University of Southern California, Los Angeles, CA 90089, USA; bryantth@usc.edu (B.T.); anoop.0112@gmail.com (A.T.)

**Keywords:** modular microfluidics, thermal sensor, flow sensor

## Abstract

A discrete microfluidic element with integrated thermal sensor was fabricated and demonstrated as an effective probe for process monitoring and prototyping. Elements were constructed using stereolithography and market-available glass-bodied thermistors within the modular, standardized framework of previous discrete microfluidic elements demonstrated in the literature. Flow rate-dependent response due to sensor self-heating and microchannel heating and cooling was characterized and shown to be linear in typical laboratory conditions. An acid-base neutralization reaction was performed in a continuous flow setting to demonstrate applicability in process management: the ratio of solution flow rates was varied to locate the equivalence point in a titration, closely matching expected results. This element potentially enables complex, three-dimensional microfluidic architectures with real-time temperature feedback and flow rate sensing, without application specificity or restriction to planar channel routing formats.

## 1. Introduction

Thermal sensing plays a vital role in chemical engineering systems by providing quantitative, non-specific monitoring of process reactions and conditions. Traditionally, devices for real-time temperature measurement are immersed in bulk quantities of reagent as they are combined in batch or continuous flow reactors. Unpredictable transport and mixing behavior of macroscopic fluid systems can affect both the predictability of processes as well as the reliability of data collected by immersed thermal probes. Micro- and millifluidic systems handling liquid reagents can eliminate artefacts in fluid handling by linearizing transport and mixing behavior, as well as taking advantage of a variety of other physical phenomena specific to low-Reynolds number flows [1,2,3,4]. However, existing thermal probes for microfluidic systems are often either highly application specific or limited to planar manufacturing formats derivative of thin-film semiconductor processing technology. One such example is the use of liquid phase temperature-sensitive indicators that produce low-resolution visible light signals, such as fluorescent dyes [5,6,7,8,9,10]. These probes are limited in their application by the potential for unintended reactivity with processing reagents and are therefore ill-suited for use as a general solution. Methods of thermal detection that do not require reaction indicators have also been demonstrated, including infrared thermography [11,12] and direct integration of electronic temperature sensors with microfluidic channels. Thermography requires specialized optical equipment and provides relatively low resolution, making it unsuitable for real-time monitoring of complex systems. It also requires direct line-of-sight to the channel being integrated, limiting system complexity. Sensor integration avoids these constraints, and devices such as thermocouples/thermopiles [13,14,15,16,17,18], thermistors [19,20,21,22], and resistance temperature detectors [23,24,25,26] have been successfully fabricated alongside microfluidic channels. These devices require specialized thin-film manufacturing practices such as micromachining that are costly and limit their format to planar design, making them better suited as instruments for purely analytical activities (e.g., microcalorimetery).

Previously, we demonstrated a standardized system of modular, reconfigurable discrete microfluidic elements useful for constructing three-dimensional circuits [27,28]. Briefly, we constructed a library of components using stereolithography that perform simple functions in microfluidic transport and measurement. This system enables engineers to construct predictable and modular miniaturized systems for process and analytical chemistry with scalable complexity. In this report, we expand our library of elements to include a thermal probe for monitoring continuous flows of process reagents or assessing unknown flow rates. The element was designed by employing the technique of integrating market-ready discrete electronic devices with our discrete microfluidic element framework. More specifically, we demonstrate a device in which we reversibly sealed a glass-bodied thermistor within a microfluidic channel element such that it was maximally submerged by inlet flows. The resulting discrete microfluidic element serves an accurate, precise, modular and low-cost temperature sensor that is deployable for in-line monitoring of temperature without limited chemical application or planar format specificity.

## 2. Results

### 2.1. Design Principle

There are several discrete electronic elements for thermal sensing commonly available on the market, each with its own unique set of advantages and disadvantages. Thermistors were selected to be integrated into our microfluidic platform for their fast response times, high sensitivity, generally good accuracy, and low cost compared to resistance temperature detectors or thermocouples. However, thermistors are not particularly linear or stable over long periods of use. For example, the thermistors used in this study have a resistance sensitivity of approximately 4.5%/°C around room temperature, a tight manufacturing tolerance of 1%, and cost less than (US) $1 when purchased in lots of 100 or more at the time of this writing. However, they also vary approximately 3.26 and 0.25 times their nominal room temperature value at 0 and 60 °C, respectively, demonstrating significant nonlinearity across a typical range of laboratory conditions. These were considered as acceptable trade-offs; a secondary objective for design of discrete microfluidic elements is to ensure they are semi-disposable and easily replaceable. Furthermore, high-precision thermistors are readily accessible, ensuring that empirical calibration procedures should be applicable over a broad number of duplicate devices.

Thermistors are internally composed of a chip of ceramic or polymer that is connected at two terminals to electrodes. The chip is then coated with relatively thermally conductive glass or epoxy, forming its external packaging. Non-specialized, glass thermistors are generally available in two packaging geometries herein dubbed “radial” and “bead”. Cylindrically shaped radial thermistors are generally larger than bead thermistors and have stiffer electrodes with tight manufacturing tolerances informed by their use in printed circuit board design. They typically have a lower surface-area-to-volume ratio than semi-spherical bead packages, implying a qualitatively more sluggish response to temperature changes. Nevertheless, their geometry is critically easier to integrate with microfluidic components that are assembled by hand, and were therefore selected as the packaging of choice in the design presented in this report.

**Figure 1:**
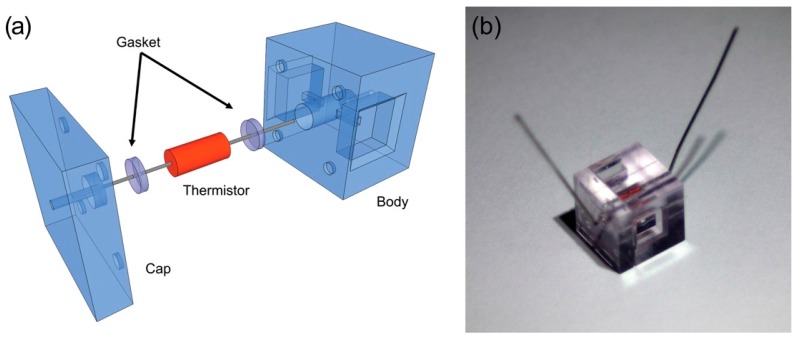
(**a**) Exploded-view Computer Aided Design (CAD) model, depicts the insertion of a radial-style thermistor inserted into a microchannel element through a single face and sealed by polydimethylsiloxane (PDMS) gaskets with two slits for electrical leads; (**b**) Assembly of the device demonstrated in this work.

The discrete microfluidic element shown in Figure 1 was constructed such that glass body of a radial thermistor was submerged in the microfluidic channel, maximizing contact area for heat conduction between fluid and the thermistor surface. This was accomplished by fabricating each component in two parts, allowing for simple, bench top level assembly. These parts, dubbed the “body” and “cap”, of the element were self-registered by a pin-and-socket interference fit similar to that used in larger assemblies of discrete microfluidic elements. Where electronic leads were drawn out of the body and cap, small silicone gaskets were inserted. Upon assembly of the cap and body, the gaskets compressed to seal unintended gaps for leaks and isolate the leads electrically (see Section 4). The combination of self-alignment between the cap and body as well as the friction between the thermistor and gasket held the thermistor approximately in the center of the microchannel element in the body. The result was a channel area roughly 200 µm wide around the thermistor packaging. No leaks were detected from this channel through the cap-body interface at continuous flow rates of deionized water as high as 50 mL/h, or roughly 0.75 kPa. A small amount of epoxy was used between the cap and body to improve the leak proofing to flow rates as high as 150 mL/h, or roughly 2.2 kPa.

### 2.2. Flow Rate Dependent Response

**Figure 2:**
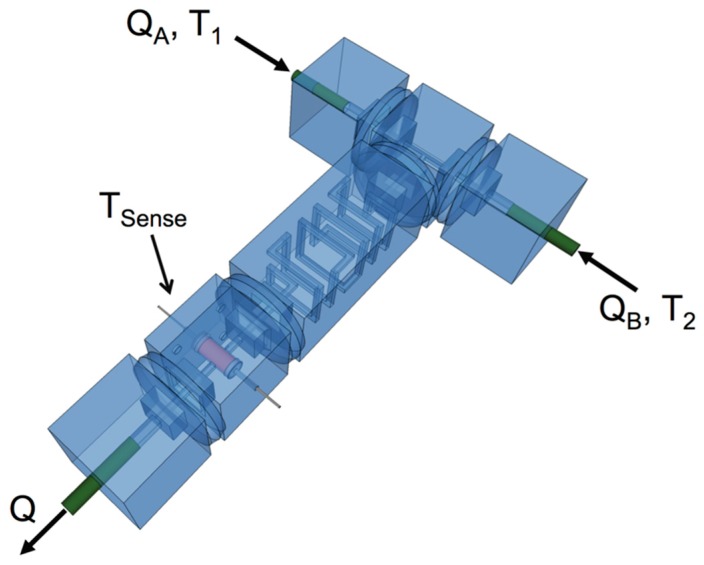
Microfluidic assembly composed of the discrete elements in Figure A1 used to perform the characterizations reported in this work. For each flow rate, the test device was allowed to reach steady state as judged by stability of the measured temperature. The temperature of the syringes and test device were then recorded over 30 s, approximately once a second. The resulting offset between the test device reading and average of the syringe readings was then computed.

Thermistors summarily behave as temperature-dependent, nonlinear resistors. Consequently, electrical current developed through a thermistor will cause Joule heating of the device. This heat can offset the absolute measurements of the temperature if the thermistor is unable to dissipate power effectively. In microfluidic channels, substantial hydraulic resistances $R_{\mathrm{hyd}}$ relative to macroscopic, traditional channels may also result in the generation of heat from viscous, laminar flows. These values are often in the MPa∙s/m^3^ to GPa∙s/m^3^ range, and for typical pressure-driven flow conditions they often result in a Reynold’s Number less than 0.1 [27,28,29]. For a device such as that shown in Figure 2 to achieve a steady-state measured temperature ($T_{m}$), a balance between heat generated due to self-heating of the thermistor and microchannels and heat dissipated by the circuit overall must be established. This is expressed mathematically by balancing power generated by an electrical resistor $P_{\mathrm{eR}}$ and hydraulic resistor $P_{\mathrm{hR}}$ with a general expression for power dissipation$\;P_{c}$. Equations (1) and (2):

(1) $$P_{\mathrm{eR}}+P_{\mathrm{hR}}=P_{c}$$

(2) $$\frac{V^{2}}{R\left(T_{f}\right)}\;+Q^{2}R_{\mathrm{hyd}}=h\left(Q\right)\left(T_{m}\left(R\right)-T_{f}\right)$$

Here, $V^{2}/R$ is the heat generated by the thermistor with resistance R as a function of its temperature $T_{f}$ due to the voltage V across its leads, nominally around 0.625 mW (or 2.5 V across 10 kΩ at 25 °C). Similarly, $Q^{2}R_{\mathrm{hyd}}$ is the heat generated by flow rate Q through the microfluidic circuit, often on the order of microwatts to milliwatts for typical assemblies. $T_{f}$ represents the actual temperature of surrounding fluid intended to be measured. In the device presented in this report, both the measured temperature $T_{m}$ and the power dissipated by the thermistor are dependent on its resistance R. h is a flow rate dependent prefactor that accounts for the ability of the fluid, surrounding packaging, and laboratory environment to transfer heat generated in the thermistor and microfluidic circuit to the laboratory environment. However, conduction of heat away from the fluid to the ambient environment from within the microfluidic circuit is expected to be minimal. This is validated by the observation that the thermal conductivity of the thermistor glass-body is an order of magnitude higher than that of the plastic used to construct discrete microfluidic elements. In addition, the thickness of the glass is an order of magnitude thinner than the thickness of the plastic. Therefore, heat is largely transferred between the thermistor and surrounding flows through forced convection, implying that h will be dependent on the flow-rate Q through the device. In addition, h is a function of the surface area of the thermistor-fluid interface, geometry, type of fluid, and other device design and operation specific parameters, which are all constant for a given experiment in this report. An analytical solution to Equation (2) is unlikely, considering the potential complex dependency of power balance on flow rate, making an empirical assessment of measurement nonlinearity the ideal approach to characterizing device response.

**Figure 3:**
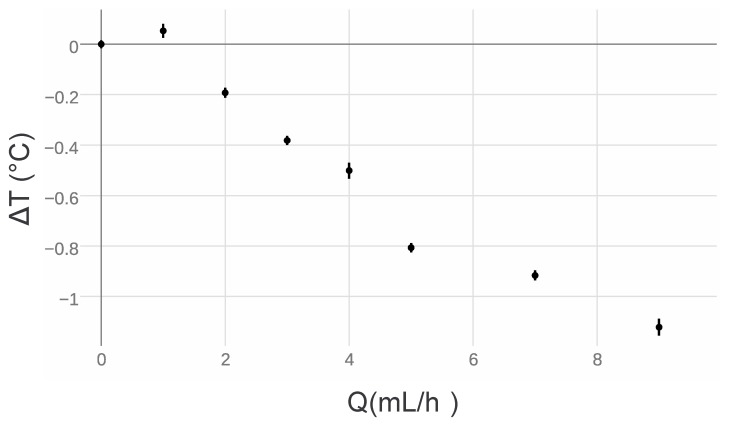
Device response with respect to flow rate. The difference ∆*T* between the temperature recorded by the device and the average syringe temperature was computed for across a range of flow rates *Q* of deionized water in the assembly shown in Figure 2. Data were shifted by the difference measured in the condition of no flow ∆*T* (0 mL/h), providing an empirical assessment of the flow rate-dependent offset between measured temperature and fluid temperature. The thermistor appears to perform with little variation to measured temperature at low flow rates. Microfluidic flow appears to cool the thermistor starting around 2 mL/h. Error bars shown represent the standard deviation of data collected during the recording period.

In applications where the device is used to measure the temperature of a microchannel flow, the flow-rate dependent offset between measured temperature and the fluid temperature is required in order to gather the most accurate result. For the device presented in this report, heat dissipation from the thermistor in typical laboratory conditions and working flow rates was found to be independent of flow rate below 2 mL/h and approximately linear thereafter (Figure 3). Figure 2 describes the experimental setup used to perform the measurement. Two syringes (A and B) with measured temperature $T_{1}$ and $T_{2}$ were used to drive deionized water through the device from a single syringe pump such that $Q_{A}=Q_{B}$. Note that in all experiments, the temperature of these syringes were left uncontrolled, reflecting ambient laboratory conditions (Figure A2 and Figure A3). The flow rate-dependent temperature measured in the test device $T_{\mathrm{sense}}\left(Q\right)$ was recorded alongside $T_{1}$ and $T_{2}$ in order to compute the temperature difference $\Delta T'=T_{\mathrm{sense}}-<T>$ where <T> is the average of the syringe temperatures at steady state. The experiment was repeated across a range of total flow rates $Q=Q_{A}+Q_{B}$. During data processing, ΔT' was shifted by its value in a stopped-flow condition ΔT' (Q=0) in order to effectively “zero-out” the difference between the syringe temperatures, experimental device temperature, and calibration errors due to intrinsic variations in their associated thermistors and self-heating conditions. This assumption was validated through the observation that the Rayleigh Number in a microfluidic channel with characteristic length of 200 µm is extremely low (approximately 0.16), indicating that free convection is insignificant in a stopped-flow condition. As shown in Figure A2 this measure ΔT = ΔT'−ΔT'(Q=0) is stable with time at all flow rates measured, indicating that the system consistently achieves steady state.

### 2.3. Continuous Flow Titration

**Figure 4:**
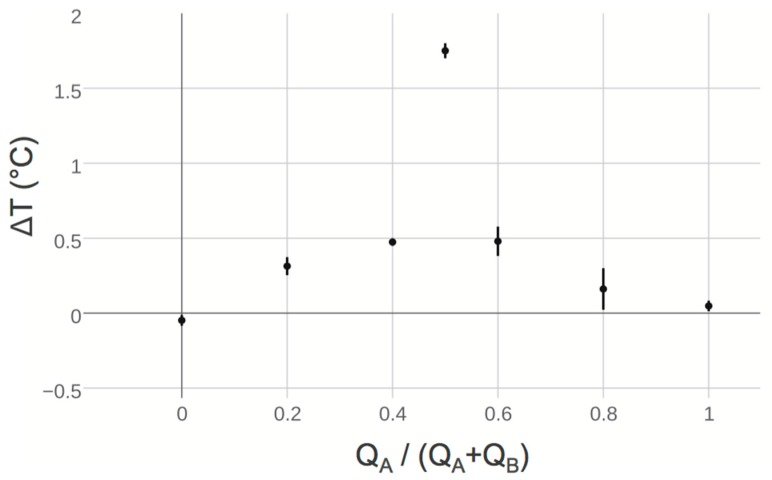
Two solutions, an acid and base, with approximately equal normality were combined in the microfluidic circuit shown in Figure 2 to perform a continuous-flow titration. Their volumetric mixing ratio *Q*_A_/(*Q*_A_ + *Q*_B_) was varied by parametrically by increasing the flow rate of acid solution while decreasing the flow rate of base solution such that the total flow rate *Q* = *Q*_A_ + *Q*_B_ in the sensor was always 5 mL/h. The change in temperature before and after mixing ∆*T* was measured in a similar manner used to characterize device flow rate dependent response, predictably locating the equivalence point at a volumetric mixing ratio 0.5. The resulting data were shifted by the average value due only to device behavior, or when only acid or base solution was present. Error bars shown represent the standard deviation of data collected during the recording period.

In order to demonstrate the ability of our device to act as an effective in-line process monitor, we performed a set of simple continuous flow neutralization reactions. The microfluidic circuit shown in Figure 2 was driven by two independent pumps with flow rates $Q_{A}$ and $Q_{B}$ representing acid and base solutions of equal concentration respectively. These solutions were combined in the T-junction, mixed through diffusion in a long length of channel, and probed with our thermistor element. The difference in steady state temperature reading before and after mixing ΔT' was computed and recorded in the same manner as used in assessing the flow-rate dependent nonlinearity of the test device. The volumetric mixing ratio $Q_{A}/\left(Q_{A}+Q_{B}\right)\;$was parametrically varied, allowing for the construction of the titration curve shown in Figure 4. The sum of their flow rates $Q_{A}+Q_{B}$ was held constant throughout the experiment so as to prevent flow-rate dependent nonlinearity from contributing unexpected offsets to individual data. Note that ΔT' was shifted by the average of offsets in a no-reaction condition, or where the volumetric mixing ratio was 0 and 1 (see Flow Rate Dependent Response). The resulting curve reflects the expected behavior of the reaction conditions, showing maximal heat release when equal volumes of acid and base solution with equivalent normality mix in the microfluidic circuit. Thus ΔT is symmetric around the volumetric mixing ratio 0.5, reflecting acid-limited and base-limited conditions in the reaction to the left and right of this value respectively.

## 3. Discussion

We have successfully demonstrated a new discrete microfluidic element for in-line thermal sensing and process management. A market-available glass-bodied thermistor with radial packaging was aligned within a microfluidic channel element using the same self-registering interference fit methods otherwise used to interconnect discrete microfluidic elements at an assembly level. The device shows good predictability in typical laboratory conditions: the flow rate dependent response is nearly linear at working flow rates, enabling potential use even as a single-ended flow rate sensor. This linearity occurs as a result of competition between power generated by the thermistor and viscous heating of surrounding microflows and power dissipation due to forced convection by surrounding microflows and to the environment. In turn, this limits device operation as a flow rate sensor to scenarios in which the steady state temperature of the fluid and sensor are unequal, which may not be the case in which the fluid is preheated or heat is internally generated by an exothermic reaction.

Furthermore, the device predictably locates the equivalence point in a continuous flow acid-base titration, showing its effectiveness at detecting changes in temperature in processes involving even low concentrations of reagents. Ultimately, this device is free of format or application specificity, and is easily deployed in complex, three-dimensional microfluidic assemblies composed of discrete elements. This approach to integration of off-the-shelf electronics in 3D-printed microfluidic elements provides a general template for the creation of microfluidic sensing and actuation modules. Such an approach can capture the full range of functionality provided by commercial microelectronics without requiring any specialized microfabrication procedures.

## 4. Materials and Methods

### 4.1. Electronic and Software Interfaces

Radial-style glass bodied NTC (Negative Temperature Coefficient) thermistors (103JG1F, U.S. Sensor Corp., Orange, CA, USA) were used for all temperature measurements in this study. Thermistor leads were connected in a voltage divider scheme to a microcontroller development board with an on-board 10-bit ADC (Arduino Mega, SparkFun Electronics, Niwot, CO, USA), as seen in the electronic schematic given by Figure A4 The internal ADC voltage was referenced to the 5 V line for convenient accurate reading of the range free of drift or ripple in the power supply. Controller software was written to read the temperature-dependent voltage drop across the thermistor, compute its resistance, and calculate the temperature using the Steinhart-Hart Equation [30]. More specifically, ten readings were taken in 1 ms intervals and averaged when the controller was commanded to return a result; the ADC measurement takes approximately 100 µs. This reading was in turn communicable over standard serial to a computer or other data recorder. Precision resistors (0.25%) were used to divide a 5V line voltage with the thermistor, though their actual values were measured to the milliohm level and stored on-board in the controller software to minimize their contribution to errors in accuracy. The divider was designed in order to provide a detectable voltage approximately half of the reference supplied to the ADC at a nominal temperature of 25 °C, ensuring a wide range of detectable temperatures. Coefficients for the Steinhart-Hart Equation were pre-computed from resistance-temperature tables provided by the thermistor manufacturer using matrix inversion and also stored on-board. The resolution of temperature readings is directly limited by the resolution of the ADC. For the device demonstrated in this study, the smallest readable change in signal is approximately 4.878 mV, corresponding to approximately 0.089 °C.

### 4.2. Microfluidic Components and Experiments

**Figure A1:**
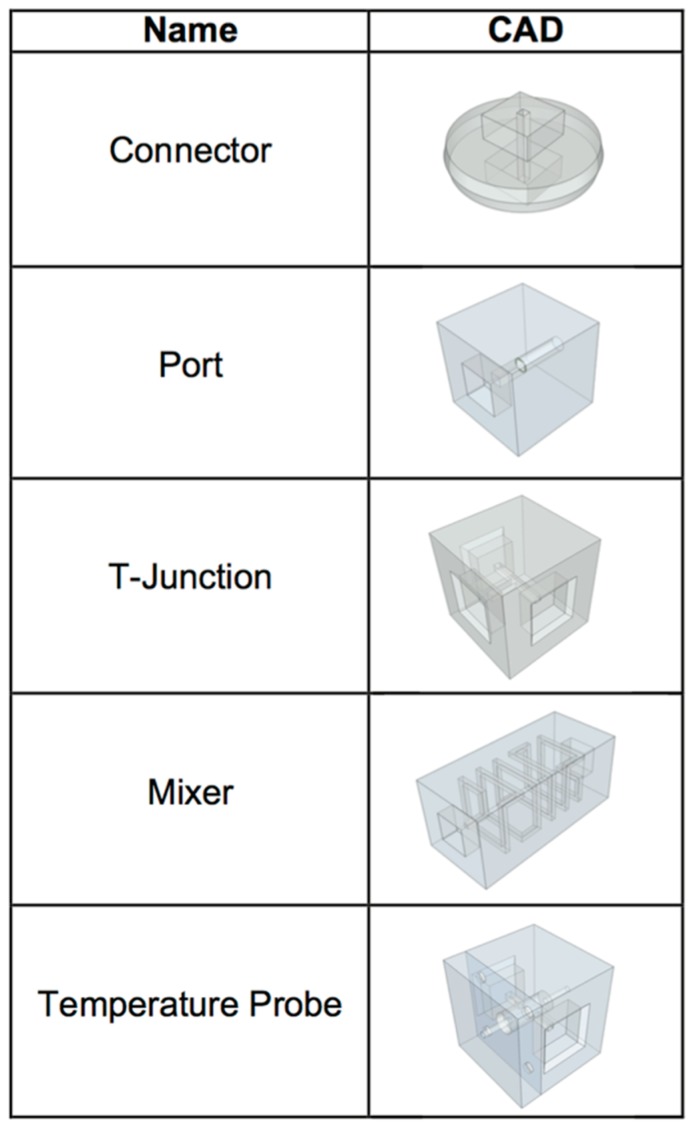
Library of discrete microfluidic elements used in this study. All channels had square cross-sections and were 642.5 µm wide (as designed). In keeping with the standard geometric constraints of discrete microfluidic elements previously demonstrated, each component was a constrained to a single cube of 1 cm side width. Connectors were 6 mm long and the mixer was 2.6 cm long, the equivalent length of two standard cubes and one connector.

**Figure A2:**
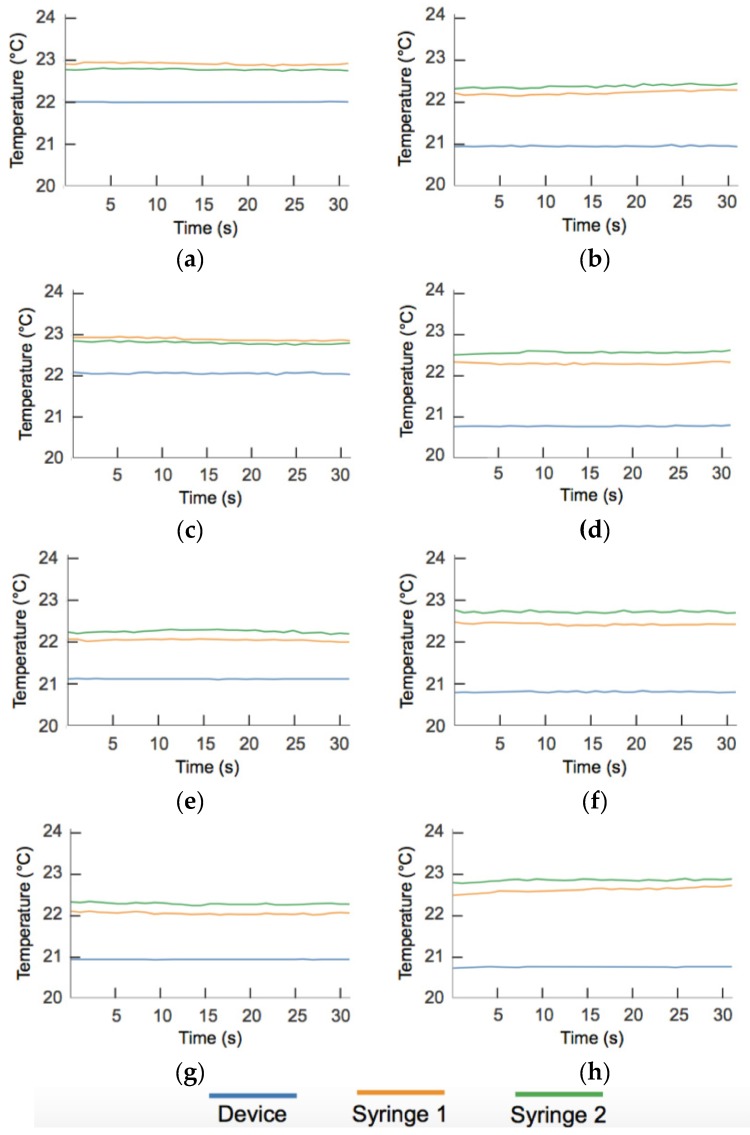
Temperature recordings over the steady state measurement period in the experiment described in Results and Discussion to measure device nonlinearity as a function of flow rate. See Section 4 for a detailed description of experimental protocol. (**a**) 0 mL/h; (**b**) 4 mL/h; (**c**) 1 mL/h; (**d**) 5 mL/h; (**e**) 2 mL/h; (**f**) 7 mL/h; (**g**) 3 mL/h; (**h**) 9 mL/h.

**Figure A3:**
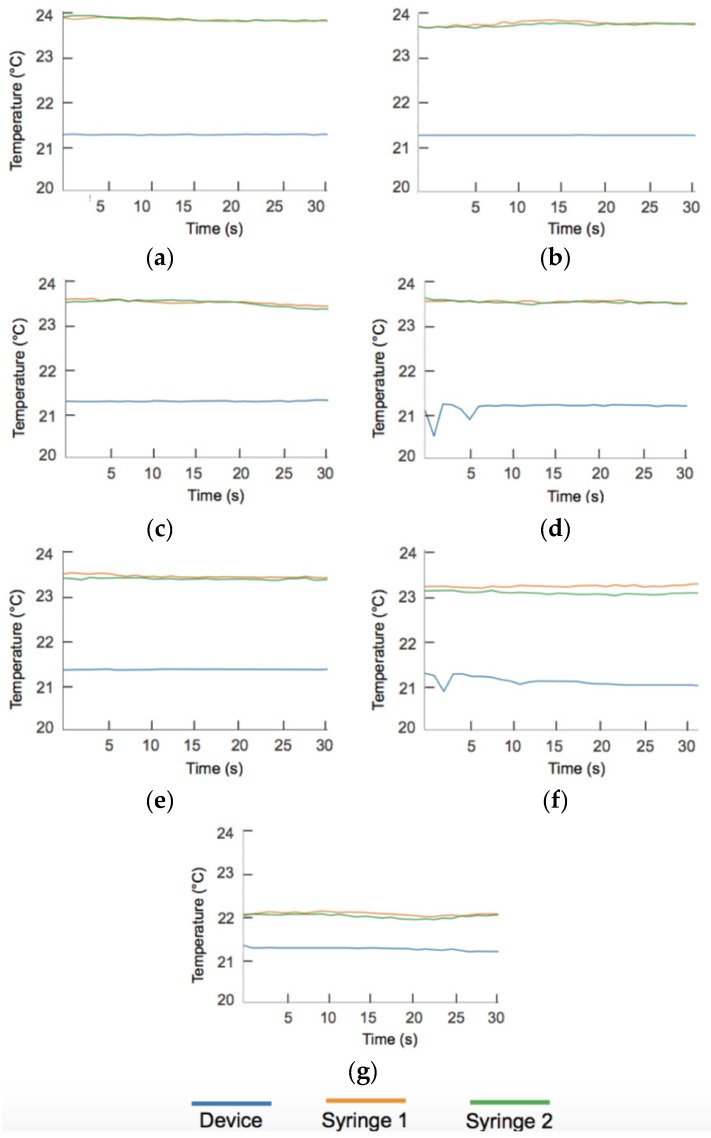
Temperature recordings over the steady state measurement period in the continuous flow titration experiment described in Results and Discussion. The equivalence point in the titration occurs in the condition $\;Q_{A}=Q_{B}$. This is seen when the difference between the average syringe temperature and the device temperature is minimal across a range of volumetric mixing ratios, reflecting maximal device heating. See Section 4 for a detailed description of experimental protocol. (**a**) *Q*_A_ = 0 mL/h, *Q*_B_ = 5 mL/h; (**b**) *Q*_A_ = 5 mL/h, *Q*_B_ = 0 mL/h; (**c**) *Q*_A_ = 1 mL/h, *Q*_B_ = 4 mL/h; (**d**) *Q*_A_ = 4 mL/h, *Q*_B_ = 1 mL/h; (**e**) *Q*_A_ = 2 mL/h, *Q*_B_ = 3 mL/h;(**f**) *Q*_A_ = 3 mL/h, *Q*_B_ = 2 mL/h; (**g**) *Q*_A_ = 2.5 mL/h, *Q*_B_ = 2.5 mL/h.

**Figure A4:**
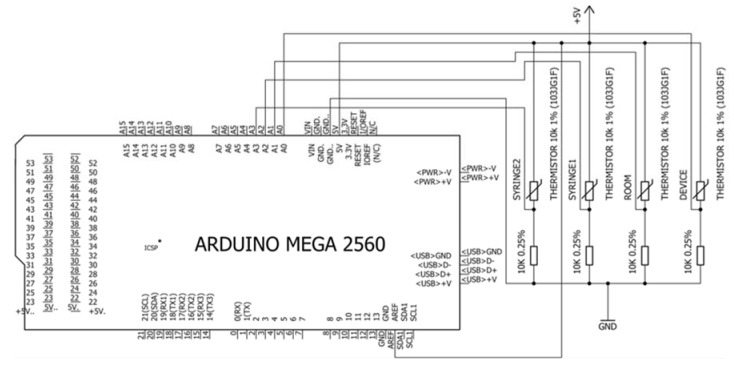
Electronic schematic of the circuit used in this study to collect thermistor data.

All microfluidic components were fabricated from transparent photoresin (Accura^®^ ClearVue Free, 3D Systems, Valencia, CA, USA) by stereolithography in a manner similar to that previously used to construct discrete microfluidic elements [27,28]. Holes were fabricated as part of the design to route thermistor electrical leads away from detectable flow areas. A thin layer of polydimethylsiloxane (PDMS) was cut using a hole punch and a razor blade to act as a gasket to seal thermistor leads away from fluid flow and prevent leaks. More specifically, a thin cut was introduced into the PDMS slab using the corner of a razor where a lead was intended to be threaded. Upon assembly of the cap and body (Figure 1), the gaskets expanded radially to form seals around the leads due to the axial compression from by-hand press fitting. A good seal was indicated by sudden optical clarity of the gasket. In some devices, a small drop of fast drying epoxy (Loctite^®^ Epoxy Instant Mix™ 5 Minute, Henkel Corp., Westlake, OH, USA) was dragged around the center pin of the cap before assembly. The library of discrete microfluidic elements utilized in this study is provided in Figure A1 The flow-rate dependent behavior of the thermal sensor elements was determined by connecting the test assembly shown in Figure 2 to a syringe pump (PHD 2000, Harvard Apparatus Inc., Holliston, MA, USA) loaded with 30 mL syringes of deionized water. Measurements were collected after allowing at least 8 minutes of steady state flow in order to thermally stabilize the system conservatively (stability in the measurement typically occurred within 1 minute and was determined as less than 1% drift over a 30 s window). Approximately 30 s of temperature data were recorded and averaged for each flow-rate variation (timing curves are provided in Figure A2). The average coefficient of variance for these measurements was 0.1565%, indicating good reliability of the timing parameters used in this experiment. The continuous-flow titration experiment was performed in the same manner, except with two independent syringe pumps (Harvard Apparatus 22, Harvard Apparatus Inc., Holliston, MA, USA), one loaded with a 30 mL syringe of acid solution and another loaded with a 30 mL syringe of base solution (timing curves are provided in Figure A3). 8 mM solutions of HCl and NaOH in deionized water were used as a model solutions of equal normality, giving the expectation that a volumetric mixing ratio of 0.5 would release the most heat. The measured pH of base solution was 11.48 (expected was 11.9) and the measured pH of acid solution was 2.05 (expected was 2.10).
